# The rod synapse in aging wildtype and *Dscaml1* mutant mice

**DOI:** 10.1371/journal.pone.0290257

**Published:** 2023-11-01

**Authors:** Mellisa R. Clemons, Ren H. Dimico, Cailyn Black, Megan K. Schlussler, Michael J. Camerino, Kirah Aldinger-Gibson, Amaris Bartle, Nathan Reynolds, Dylan Eisenbrandt, Aspen Rogers, John Andrianu, Bradley Bruce, Arthur Elliot, Tom Breazeal, Hannah Griffin, Molly K. Murphy, Peter G. Fuerst

**Affiliations:** 1 Department of Biological Sciences, University of Idaho, Moscow, Idaho, United States of America; 2 Natural Sciences, North Idaho College, Coeur d’Alene, Idaho, United States of America; 3 WWAMI Medical Education Program, University of Washington School of Medicine, Moscow, Idaho, United States of America; 4 Department of Biochemistry, Wake Forest School of Medicine, Winston Salem, North Carolina, United States of America; Universidade Federal do ABC, BRAZIL

## Abstract

The retina is an intricately organized neural tissue built on cone and rod pathways for color and night vision. Genetic mutations that disrupt the proper function of the rod circuit contribute to blinding diseases including retinitis pigmentosa and congenital stationary night blindness (CSNB). Down Syndrome cell adhesion molecule like 1 (*Dscaml1)* is expressed by rods, rod bipolar cells (RBCs), and sub-populations of amacrine cells, and has been linked to a middle age onset of CSNB in humans. However, how *Dscaml1* contributes to this visual deficit remains unexplored. Here, we probed *Dscaml1’s* role in the maintenance of the rod-to-RBC synapse using a loss of function mouse model. We used immunohistochemistry to investigate the anatomical formation and maintenance of the rod-to-RBC synapse in the young, adult, and aging retina. We generated 3D reconstructions, using serial electron micrographs, of rod spherules and RBCs to measure the number of invaginating neurites, RBC dendritic tip number, and RBC mitochondrial morphology. We find that while rod-to-RBC synapses form and are maintained, similar to wildtype, that there is an increase in the number of invaginating neurites in rod spherules, a reduction in RBC dendritic tips, and reduced mitochondrial volume and complexity in the *Dscaml1* mutant retina compared to controls. We also observed precocious sprouting of RBC dendrites into the outer nuclear layer (ONL) of the *Dscaml1* mutant retina compared to controls. These results contribute to our knowledge of *Dscaml1’s* role in rod circuit development and maintenance and give additional insight into possible genetic therapy targets for blinding diseases and disorders like CSNB.

## Introduction

The neural retina is composed of two connected circuits: one for color vision, and the other for vision in dim light, or night vision. Some of the common causes of night vision loss (nyctalopia), including vitamin A deficiencies and cataracts, are readily treatable. Nyctalopia resulting from genetic mutations, including those that cause retinitis pigmentosa (RP) and congenital stationary night blindness (CSNB), have been historically more difficult to treat but are current targets for successful gene therapy approaches to restore the target gene function and prevent blindness [1–8]. Examples include X-linked CSNB and Leber congenital amaurosis (LCA), caused by mutations in *NYX* and *GUCY2D* (respectively), where patients suffer a decreased ability to see in dimly lit environments that is usually present at birth or develops during adolescence [9–11]. The success of gene therapy approaches in treating genetic causes of vision loss has highlighted the need to identify and better understand the activity of genes linked to blinding diseases in order to expand the pool of candidates for gene therapy trials.

*Dscaml1* has been identified as a candidate gene in humans for a middle age of onset of CSNB [12]. *Dscaml1* is expressed by and required for normal organization of several cell types in the rod pathway including rod bipolar cells (RBCs) and All amacrine cells. However, ERG studies, gross morphology and synaptic markers suggest that the rod circuit remains functional in a *Dscaml1* loss of function mouse model [13]. Studies in mice have shown that wildtype cells and synapses of the rod circuit undergo significant age-related changes including denervation at the rod synapse and ectopic sprouting of horizontal and bipolar cell dendrites [14–16]. We hypothesize that the middle age of onset of CSNB associated with a mutation of *Dscaml1* could reflect increased sensitivity to normal aging of the rod circuit, and here test if signs of premature or exaggerated aging are observed in *Dscaml1* mutant mice. We therefore used a *Dscaml1* loss of function mouse model to test the role of *Dscaml1* in establishing precise circuitry of the rod pathway and in maintaining the rod circuit in the aging mouse.

*Dscam* and *Dscaml1* are neural cell adhesion molecules in the immunoglobulin superfamily [17]. Dscams work alongside other cell adhesion molecules to pattern the retina, which is largely organized by differential expression of transcription factors and the adhesion molecules these regulate, as opposed to the neural activity that plays a prominent role in organizing neurons in other regions of the brain [18–21]. Mammalian Dscams play important roles in the spatial arrangement of neural soma and dendrites in bipolar, amacrine and retinal ganglion cells, and also function as a pro-growth factor in the axon pathfinding of retinal ganglion cells into the brain [22–25]. *Dscaml1* is expressed by rods, RBCs and sub-populations of amacrine cells, including AII amacrine cells, which convey visual information from RBCs to retinal ganglion cells. These cells make up the core of the rod pathway, which initiates when rods detect photons and send visual information to RBCs at the rod-to-RBC synapse. While *Dscaml1* regulates dendritic arborization and soma spacing of RBCs and AII amacrine cells, the rod-to-RBC synapse itself is histologically and functionally intact in the *Dscaml1* loss of function mouse model, as measured by immunohistochemistry, electron microscopy and ERG [13].

Here we build on previous work by utilizing a loss of function allele of *Dscaml1* to measure the requirement of *Dscaml1* for maintenance of the rod-to-rod bipolar cell synapse. We used immunohistochemistry to investigate the development and maintenance of this synapse. We observed similar patterns of retinal maturation in the wildtype and *Dscaml1* mutant retina. RBC dendrite sprouting into the outer nuclear layer (ONL), a correlation of aging and synaptic dysfunction, was increased at earlier ages in the *Dscaml1* mutant retina compared to wildtype, but similar at advanced ages. Electron micrograph studies revealed an increase in the number of neurite tips invaginating into *Dscaml1* mutant rods, a decrease in the number of *Dscaml1* RBC dendritic tips and an increase in the number of RBC dendrites that did not contact rod spherules. In summary, we report that anatomical changes were observed comparing organization of RBC dendrites of the wildtype and *Dscaml1* mutant retina, alongside subtle signs of precocious aging.

## Methods

### Animals/Housing

**Fig 1:**
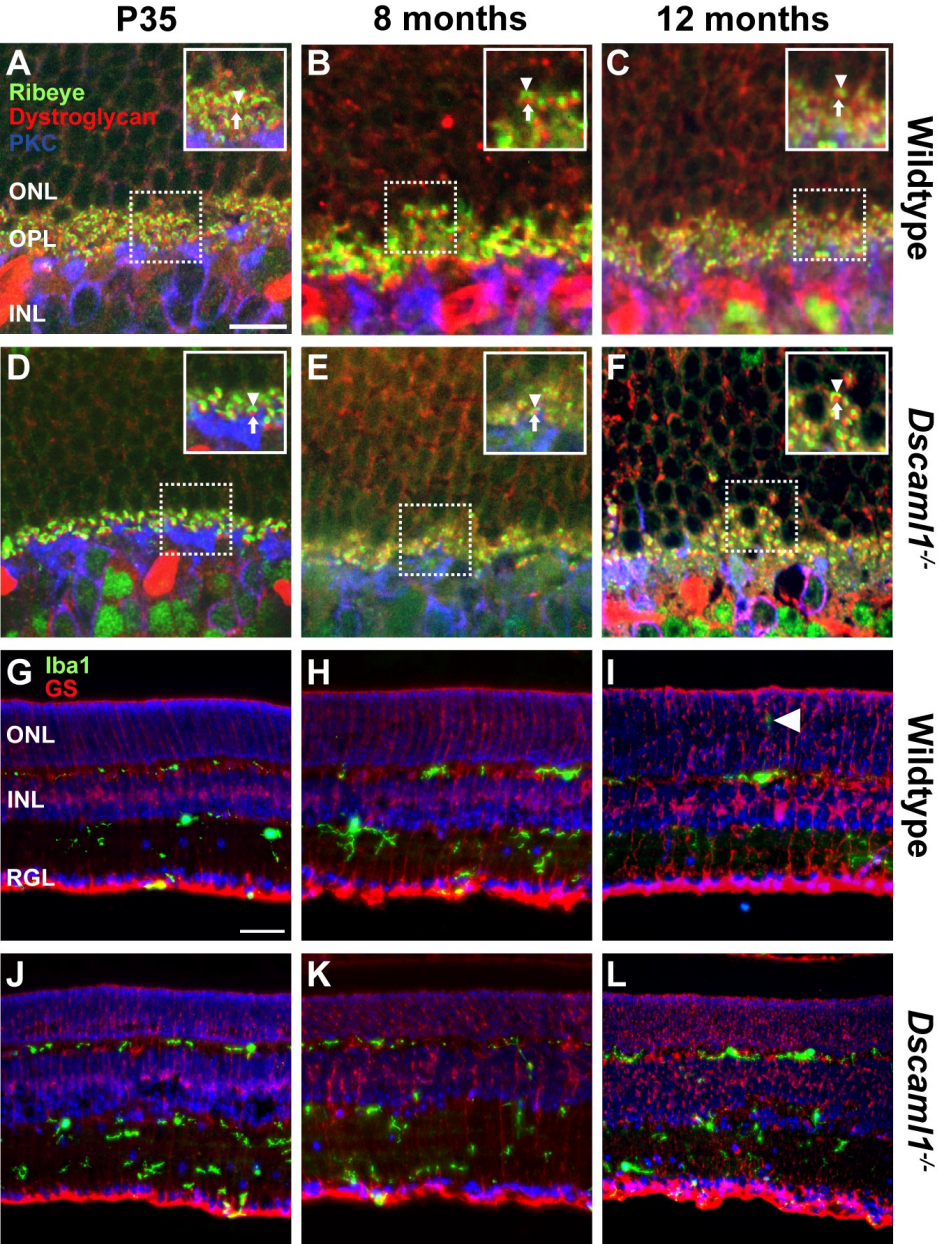
The rod-to-RBC synapse across the mouse lifespan. **A)** Representative image of rod-to-RBC synapses formed in the wildtype retina at P35. **B)** Rod-to-RBC synapses were maintained in the wildtype retina at 8 months. **C)** Rod-to-RBC synapses remain intact in the wildtype retina at one year. **D)** Representative image of synapses between rod and RBCs in the *Dscaml1* mutant retina at P35. **E)** Rod-to-RBC synapses were maintained in the *Dscaml1* mutant retina at 8 months. **F)** Rod-to-RBC synapses remain intact in the *Dscaml1* mutant retina at one year. No differences were detected comparing wildtype and *Dscaml1* mutant retinas across all the ages examined (P14, P21, P35, 3 months, 8 months, 12 months, 18-months). **G)** Representative image of Müller glia and microglia in the wildtype retina at P35. **H)** Representative image of Müller glia and microglia in the wildtype retina at 8 months. **I)** Representative image of Müller glia and microglia in the wildtype retina at one year demonstrating occasional microglia projecting into the ONL (arrowhead). **J)** Representative image of Müller glia and microglia in the *Dscaml1* mutant retina at P35. **K)** Müller glia and microglia in representative image of the *Dscaml1* mutant retina at 8 months. **L)** Representative image of Müller glia and microglia in the *Dscaml1* mutant retina at one year. No differences were observed comparing wildtype and *Dscaml1* mutant retinas. Arrowheads (A-F) point to ribeye horseshoe. Arrows (A-F) point to dystroglycan puncta. PKC = rod bipolar cells, dystroglycan = synaptic cleft, ribeye = presynaptic, GS = Müller glia, Iba1 = microglia. Abbreviations: RBC = rod bipolar cell, ONL = outer nuclear layer, OPL = outer plexiform layer, INL = inner nuclear layer. Sample size = 12 to 20 images, across at least 3 mice per genotype at all time points. Scale bar = 20 μm (A-F) and 50 μm (G-L).

**Fig 2:**
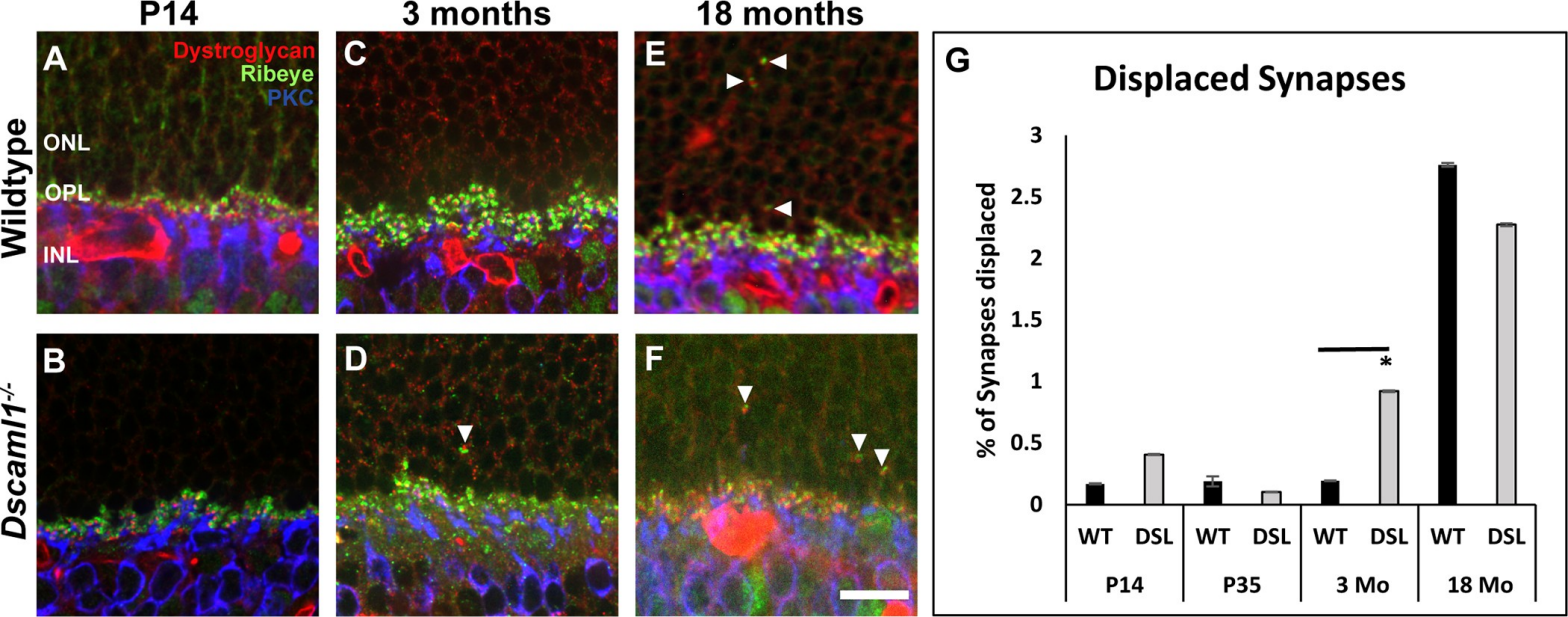
Displaced rod-to-RBC synapses during development and aging. **A)** Representative image of rod-to-RBC synapses in the wildtype mouse retina at P14. Synapses are restricted to the OPL. **B)** Rod-to-RBC synapses in the *Dscaml1* mutant retina are also restricted to the OPL at P14. **C)** Rod-to-RBC synapses in the wildtype mouse retina at 3 months. Synapses are mostly located within the OPL. **D)** An increase in the number of rod synapses was observed in the ONL of the *Dscaml1* mutant retina at 3 months of age. **E)** An increase in the number of rod synapses was observed in the ONL of the wildtype retina at 18-months. **F)** An increase in the number of displaced rod synapses was also observed in the *Dscaml1 mutant* retina at 18-months. **G)** No significant difference in the number of displaced synapses was detected comparing the wildtype, heterozygous mutants, and *Dscaml1* mutant retina at P14, P35, and 18-months (P14, p = 0.22; P35, p = 0.39; 18-months, p = 0.35). An increase in displaced synapses was detected at 3 months of age comparing the homozygous *Dscaml1* mutant retina to heterozygous mutant and wild type retina (p = 0.001 and p = 0.002, respectively). PKC = RBCs, dystroglycan = postsynaptic, ribeye = presynaptic. Abbreviations: RBC = rod bipolar cell, PKC = protein kinase C, ONL = outer nuclear layer, OPL = outer plexiform layer, INL = inner nuclear layer, WT = wildtype, DSL = *Dscaml1* mutant. Sample sizes = between 10 and 15 images, collected from 3 mice per genotype at all time points, except at 18-month heterozygous mutant, which only had 6 images across 2 mice. Total synapses counted: wildtype = 14,417 synapses, littermate control = 9,907 synapses, *Dscaml1* mutant = 10,879 synapses. Error bars = standard deviation. Scale bar = 10 μm.

Mice were housed in the University of Idaho Laboratory Animal and Research Facility (LARF). Mice were fed ad libitum and on a 12-hour light:dark cycle. *Dscaml1* mutant mice were derived from ES cell line CC0772 [13]. *Dscaml1* mutant mice carry a LacZ gene trap in the third exon of *Dscaml1* that intercepts and truncates the *Dscaml1* transcript early in the N-terminus of the protein [13]. *Bax* null mice were obtained from the Jackson Laboratory [26]. Mice were maintained on a mixed genetic background including C57BL/6J and 129/SvJ. Wild type litter mate controls were used for EM studies and in Fig 1. In Fig 2 we utilized both *Dscaml1* mutant heterozygous litter mate controls and inbred C57BL/6J mice wild type controls, the latter because our aging wild type mice succumbed before the time of study. The mice are broken out into separate pools and no difference was detected comparing litter mate heterozygous controls and C57BL/6J controls. Wild type C57BL/6 mice at 12 and 18-month of age were obtained from the NIH aged rodent center and were from a C57BL/6JN genetic background, which are C57BL/6J mice acquired, bred and periodically refreshed by the NIH from stock acquired from The Jackson Laboratory. These mice differ from the NIH strain C57BL/6N, which carries the *rd8* retinal degeneration mutation. Younger wild type mice were bred in house derived from C57BL/6J mice acquired from the Jackson Laboratory. All protocols were approved by the University of Idaho Institutional Care and Use Committee.

### Tissue preparation

Mice taken for immunohistochemistry studies were anesthetized with tri-bromoethanol, and transcardially perfused with 1X phosphate buffered saline (PBS) (10X PBS: distilled H_2_O, 80g NaCl, 2.0g KCL, 26.8g Na_2_HPO_4_ 7H_2_O, 2.4g KH_2_PO_4_, pH 7.4). Eyes were removed and hemisected in 1XPBS. The posterior eye cup was fixed in 4% paraformaldehyde (PFA) for thirty minutes at room temperature followed by three 10-minute washes in 1XPBS. Fixed tissue was sunk in 30% sucrose overnight followed by a 50/50 mixture of 30% sucrose and optimal cutting temperature (OCT; Sakura® Finetek Inc.) and frozen in liquid nitrogen. Eye cups were sectioned on a *Leica GM 1510 S* cryostat in 10 μm sections and stored in at -20°C for immunohistochemistry. All procedures were approved by the University of Idaho Animal Care and Use Committee. Mice were taken for study at post-natal days (P)14, P21, P35, 3 months, 8 months, 12 months, and 18-months.

### Serial election micrographs

Serial block-face electron micrograph (EM) images were generated by Renovo neural EM services (Cleveland, Ohio) using a Zeiss Sigma VP scanning electron microscope. Renovo protocols were followed for tissue processing. Two wildtype, two *Bax* null, and three *Dscaml1* mutant samples from two mice were used to generate electron micrograph volumes. Mice were taken for study at three months of age. Mice were perfused with cacodylate buffer and retinas were fixed for four days in cacodylate buffer before processing. Retina pieces were collected mid-range between central and peripheral retina. Sections were imaged at resolutions between 6.5–7 nm across images with 60–65 nm steps in between slices, depending on the individual data set.

### 3D electron micrograph reconstruction rendering/Data collection

The National Institutes of Health software, FIJI/ImageJ, was used for developing serial images into 3D stacks of retina tissue, using TrakEM plugin software as previously described [26]. Individual rod spherules, RBCs, and mitochondria were traced on individual area lists to enable isolated analysis. Data sets include wildtype, *Bax* null, and *Dscaml1* mutant retinas. For rod spherules, the number of invaginations were counted for each observed invagination of single neurites from interneurons (bipolar and horizontal cells). For RBC dendrite tip data collection, entire RBCs were traced on a single area list and each dendrite tip was then manually followed until it either terminated or contacted/invaginated into a rod spherule. The number of RBC dendrite tips that contacted a rod spherule was divided by the total number of dendrite tips to determine the percentage of tips that contacted rod spherules for comparison across genotypes. Mitochondria data was collected in the same manner as RBC data. Individual whole mitochondria were manually traced on individual area lists within already identified and traced RBCs. If mitochondria were not able to be traced completely due to going outside of the image stack, or were more than fifty percent contained in the axon, they were not included in the data.

### Immunohistochemistry

Retina sections were rehydrated in 1XPBS for 10 minutes, followed by incubation in blocking solution: PBS, 5% normal donkey serum, 0.1% triton for 20 minutes at room temperature. Primary antibodies were added to 0.1% block and incubated on sections overnight at 4°C. Sections were washed 3 x 10 minutes, followed by adding secondary antibodies diluted in 0.1% blocking solution and incubated for two hours at room temperature. Finally, a 1:100,000 dilution of DAPI 10 mg/ml in DMSO was added to the second of three final washes in 1XPBS, followed by coverslip mounting in 80% glycerol. Primary antibodies used: protein kinase C alpha (PKCα; anti-mouse, 1:100, Santa Cruz Biotechnology, MC5: sc-80), ribeye (anti-rabbit, 1:500, Synaptic Systems, #192 003), dystroglycan (Mouse IgG_1_, Developmental Studies Hybridoma Bank, mandag2 clone 7D11), glutamine synthase (Mouse IgG_2a_, 1:2000, Millipore, #MAB302), ionized calcium binding adapter molecule 1 (Iba1; rabbit, 1:500, WAKO Pure Chemical Industries, Ltd., #019–19741). All secondary antibodies were purchased from Jackson ImmunoResearch and used at 1:1000 dilutions.

### Imaging/Analysis

Image z-stacks of retina sections for rod-to-RBC synapse analysis were obtained from a Nikon spinning disc microscope at the University of Idaho Imaging Core. Image adjustments were made uniformly across whole images. For displaced synapse data collection, all synapses co-labelled with ribeye and dystroglycan antibodies were counted by hand, in a single in focus z-plane, including one image on either side of z-stack to accommodate for tissue not lying flat. Synapses were determined to be displaced if they were inset beyond one rod spherule into the outer nuclear layer. The number of displaced synapses was divided by the number of total synapses to determine the percent of total synapses that are displaced for each image and data presented are the means.

### Statistical analysis

Kruskal Wallis and Dunn’s post hoc test was used for comparisons of displaced synapses across ages and comparing genotypes within ages. One-way ANOVA and Tukey Kramer post hoc tests were used for comparisons of total dendrite number and mitochondrial volume. Kruskal Wallis and Dunn’s post hoc tests were used for comparison of the percent of dendrites that terminate at a spherule, mitochondrial complexity, and for percent of total mitochondria that fall over a certain volume across genotypes.

## Results

### Preserved integrity of the rod-to-RBC synapse in *Dscaml1* mutant retina in the young, adult, and aging retina

We first investigated the integrity of the rod-to-RBC synapse from post-natal development to advanced age of the retina (18-months) in wildtype and *Dscaml1* mutant mice. Different species have different age-related sensitivities to aging of the rod circuit. For example, humans exhibit loss of rods and RBCs with aging [27–30]. Denervation of the rod synapses and sprouting of RBC and horizontal cell neurites is observed in both mouse and human [15, 16, 31], while neither of these changes is observed in marmoset [32]. Several factors contributing to rod denervation associated with aging have been identified including increases in AMPK signaling and microglial activation [33, 34]. We hypothesized that loss of *Dscaml1* could increase sensitivity of the rod circuit to aging. We therefore imaged integrity of the rod synapse and associated cells, RBC dendrite sprouting and glial activation in wildtype and *dscaml1* mutant mice at time point between P14 and 18-months.

We labeled the rod-to-RBC synapse by staining retina sections with antibodies to ribeye and dystroglycan, markers of the ribbon synapse and perisynaptic space, respectively (**Fig 1A**) [35, 36]. We imaged sections in the central retina approximately halfway between the optic nerve and peripheral retina. Synapses formed and were anatomically intact as evidenced by the presence of the ribbon protein ribeye and synaptic cleft protein dystroglycan localized to the dendritic tips of RBCs, identified with PKC staining by postnatal day 14 in both wildtype and *Dscaml1* mutant retinas (not shown). Pairing was observed at RBC dendritic tips at all ages measured with no qualitative differences detected comparing wildtype and *Dscaml1* mutant retina between postnatal days 14 to 18-months of age (P35, 240, and 365 shown) (**Fig 1A–1F**). Müller glia and microglia play important roles in responding to damage in the retina and their activation and remodeling before during and after degeneration of rods [37–39]. No differences were observed in Müller glia, labeled with glutamine synthase, or microglia, labeled with antibodies to Iba1 (**Fig 1G–1L**), with occasional microglial projections into the outer nuclear layer (ONL) observed in both wildtype and *Dscaml1* mutant retina sections (Fig 1G arrowhead).

### Precocious increase in displaced rod-to-RBC synapses in the *Dscaml1* mutant retina

Previous studies have demonstrated an increase in the number of rod-to-RBC synapses in the outer nuclear layer during the aging of the neural retina [14–16]. The increase in the number of displaced synapses has also been observed in models of synaptic dysfunction and involves sprouting of RBC and horizontal cell neurites into the ONL to form synapses with denervated rod cells [16, 40–43]. We measured the number of displaced synapses in *Dscaml1* homozygous mutant, *Dscaml1* heterozygous mutant and wildtype retinas at P14, P35, 3 months, and 18-months of age compared this to the number of synapses in the outer plexiform layer (**Fig 2A–2F**). We observed an increase in the number of displaced synapses at 18-months of age in all genotypes compared to P14, P35, and 3 months. The percent of displaced synapses increases from 0.167 percent of all synapses (P14) to 2.67 percent (18-months) in wildtype, from 0.23 percent (P14) to 3.75 percent (18-months) in heterozygous mutant mice, and from 0.40 percent (P14) to 2.21 percent (18-months) in the *Dscaml1* mutant retina (p < 0.001 for all) (**Fig 2G**). We did not observe a significant difference in the number of displaced synapses comparing wildtype, heterozygous mutant, and *Dscaml1* mutant retinas at early and late stages (P14: p = 0.22, P35: p = 0.39, 18-months: p = 0.35). We did observe an increase in the percent of displaced synapses in the *Dscaml1* mutant retina compared to wildtype and heterozygous mutant controls at 3 months of age (3 months: p = 0.002 and p = 0.001, respectively) (**Fig 2G**).

### *Dscaml1* mutant rod spherules contain an increased number of invaginating neurites

**Fig 3:**
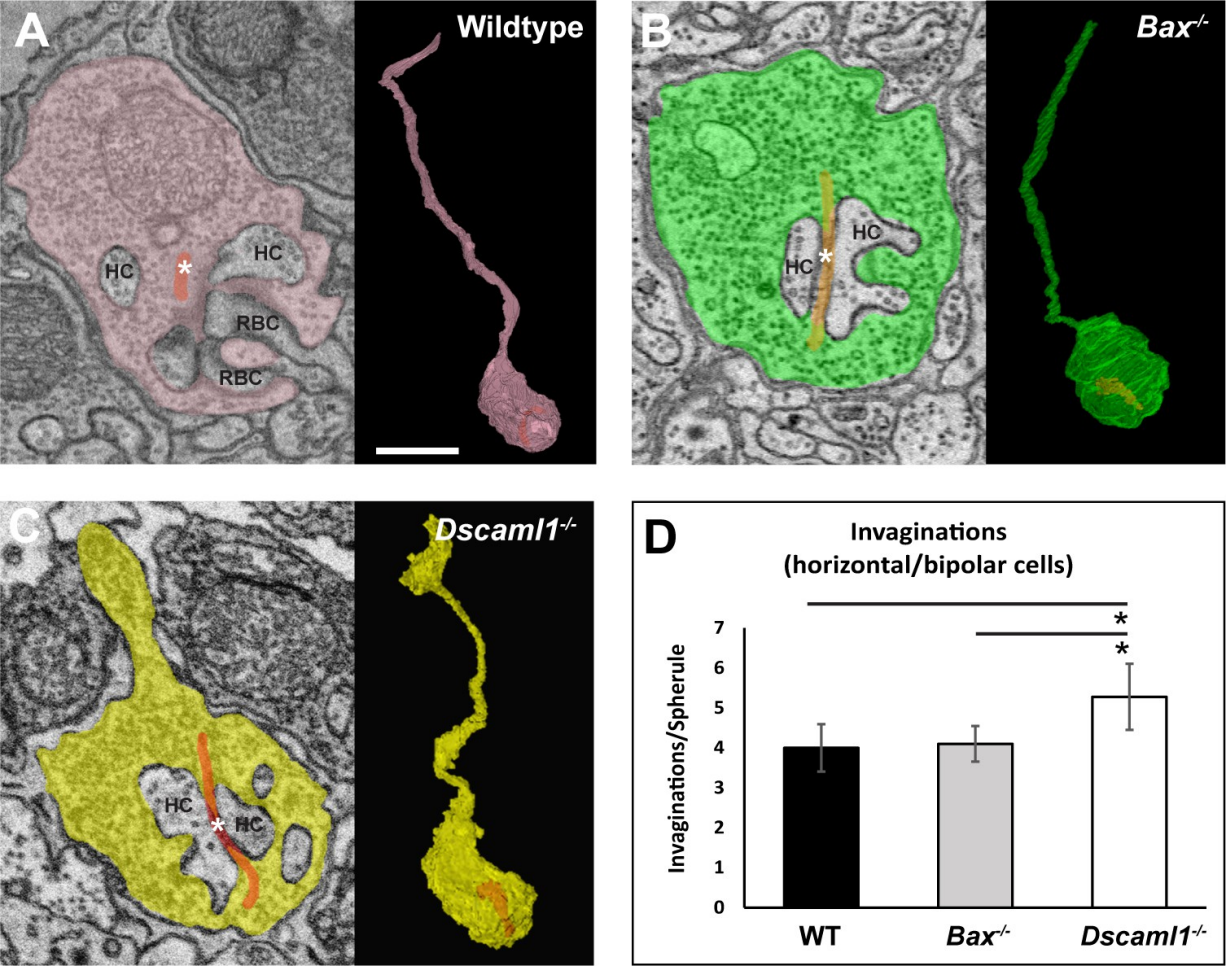
3D reconstruction of rod spherules comparing neurite invagination number. **A)** Electron micrograph and 3D rod spherule reconstruction in wildtype retina. **B)** Electron micrograph and 3D rod spherule reconstruction in *Bax* null retina. **C)** Electron micrograph and 3D rod spherule reconstruction in *Dscaml1* mutant retina. **D)** An increase in the number of neurite invaginations in *Dscaml1* mutant rod spherule was observed compared to both the wildtype and *Bax* null retinas (p < 0.001). Abbreviations: WT = wildtype, Asterisks on images = synaptic ribbon, HC = horizontal cell. RBC = rod bipolar cell. Asterisks on graph indicate significance. Spherule sample sizes = WT: 18, *Bax*: 20, *Dscaml1*: 18. Error bars = standard error of the mean. Scale bar = 5 μm.

The rod spherules, or axon terminals of rod photoreceptors, are the synaptic junction for rods, horizontal cell axon tips, and RBC and OFF bipolar cell dendritic tips [44–50]. We generated 3-D reconstructions of rod spherule electron micrographs to study the ultrastructure of *Dscaml1* mutant and control synapses. We included *Bax* null retinas as a control for RBC cell density. *Bax* null mice have an increase in the number of RBCs, but not rods, like that reported in the *Dscaml1* mutant retina [13, 51, 52]. After reconstructing the rod spherules, we compared the number of invaginations into rod spherules in wildtype, *Bax* null, and *Dscaml1* mutant retinas (**Fig 3A–3C**). Wildtype and *Bax* null spherules had no difference in the number of invaginations with an average of 4.0 and 4.1 invaginations per spherule, respectfully. *Dscaml1* spherules had an average of 5.29 invaginations per spherule compared to wildtype and *Bax* null retinas (p < 0.001 for both) (**Fig 3D**).

### Intermingling of RBC dendritic arbors in the wildtype and *Dscaml1* mutant retina

**Fig 4:**
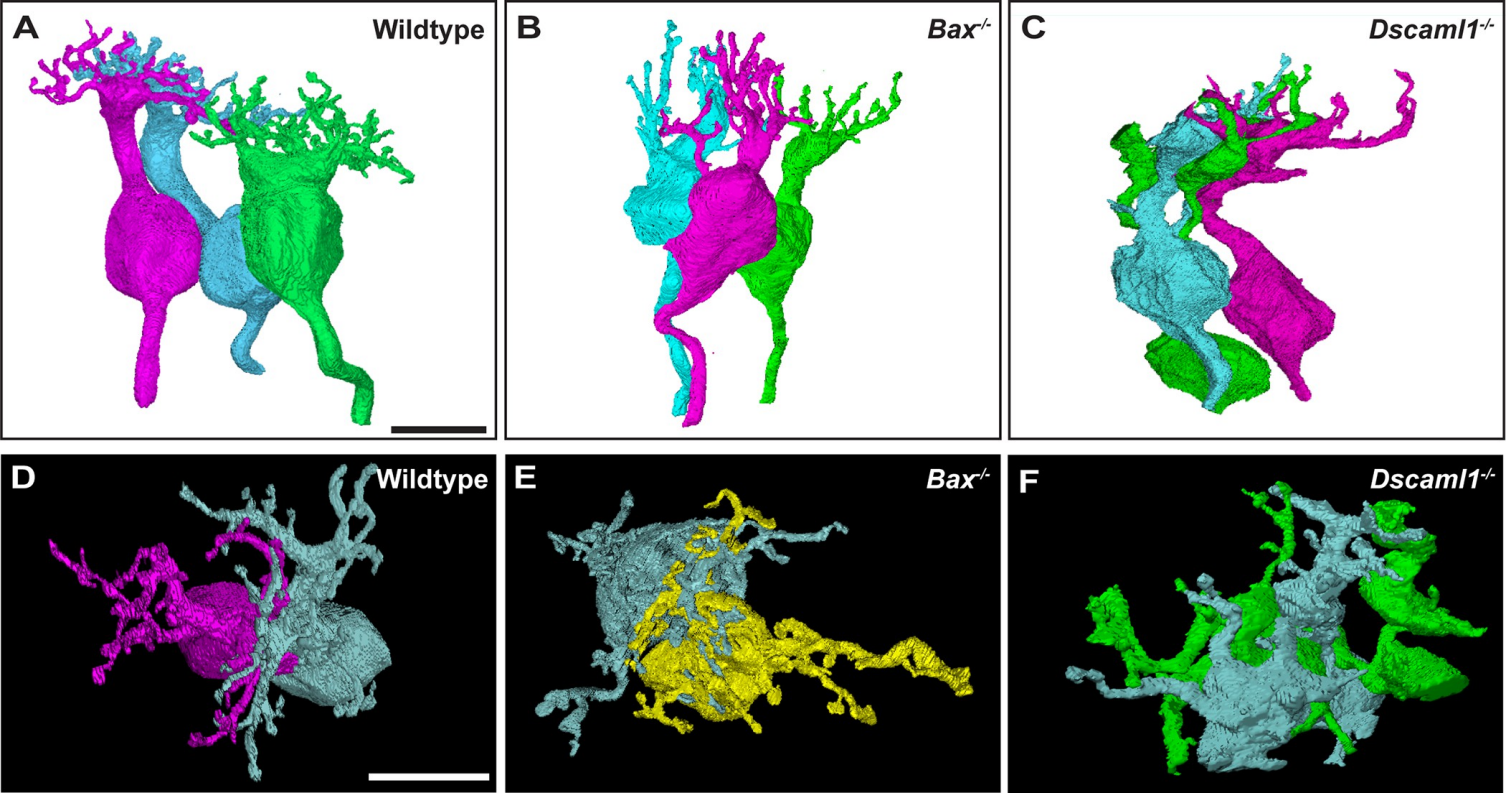
Dendritic overlap of 3D reconstructed rod bipolar cells. **A)** Representative 3D reconstructions of three neighboring RBCs’ spatial arrangement in wildtype retina. **B)** Representative 3D reconstructions of three neighboring RBCs’ spatial arrangement in the *Bax* null retina. **C)** Representative 3D reconstructions of three neighboring RBCs’ spatial arrangement in the *Dscaml1* mutant retina. **D)** 3D RBC reconstruction comparing enface dendritic area with neighboring RBC in wildtype retina. **E)** 3D RBC reconstruction comparing enface dendritic area with neighboring RBC in *Bax* null retina. **F)** 3D RBC reconstruction comparing enface dendritic area with neighboring RBC in *Dscaml1* mutant retina. Scale bars = 5 μm.

RBC dendrites do not tile, and the dendritic arbors of adjacent cells normally overlap with each other in the mouse retina [53, 54]. We reconstructed multiple RBCs to compare the RBC dendritic arbors in wildtype, *Bax* null and *Dscaml1* mutant retina (**Fig 4A–4C**). We utilized reconstructed RBCs with adjacent cell soma to compare overlap of dendritic arbors in the wildtype, *Bax* null, and *Dscaml1* mutant retina. RBC dendritic arbors in the wildtype, *Bax* null, and *Dscaml1* mutant RBCs interdigitate extensively, with distinct arbors of *Dscaml1* mutant RBC dendrites observed, compared to extensive fasciculation observed in other *Dscam or Dscaml1* deficient retinal cell types (**Fig 4D–4E**).

### *Dscaml1* mutant RBCs have a reduced number of dendritic tips and a reduced percentage of tips that terminate at rod spherules

**Fig 5:**
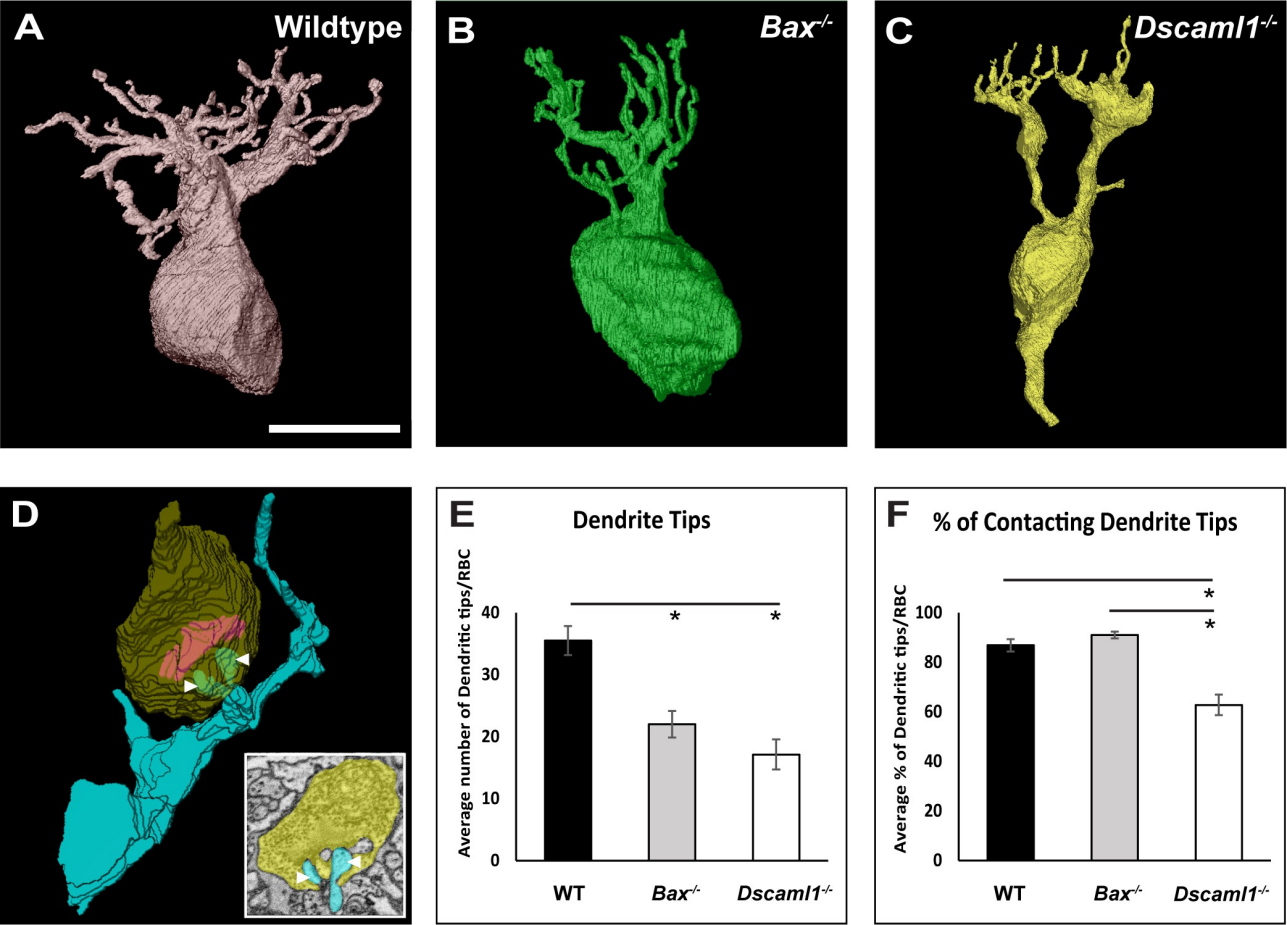
3D RBC reconstructions and dendritic comparisons. **A)** 3D RBC reconstruction in the wildtype retina. **B)** 3D RBC reconstruction in the *Bax* null retina. **C)** 3D RBC reconstruction in the *Dscaml1* mutant retina. **D)** 3D RBC dendritic tip reconstruction in *Dscaml1* mutant retina with two dendritic tips (arrowheads) terminating at the reconstructed rod spherule. Inset in D is a single slice within the serial electron micrograph stack demonstrating two RBC dendritic tips that split from a single RBC tip at point of invagination (yellow = spherule, blue = RBC dendrite tips). **E)** A decrease in the average total number of dendrites per RBC was observed in both *Bax* null and *Dscaml1* mutant retinas when compared to the wildtype retina. (WT:*Bax*, *p* = 0.019; WT:*Dscaml1*, p < 0.001; *Bax*:*Dscaml1*, p = 0.37). **F)** A decrease the percent of dendritic tips per RBC that terminate at the rod spherules in *Dscaml1* mutant retinas was observed compared to wildtype and *Bax* null retinas (WT:*Bax*, p = 0.350; WT:*Dscaml1*, p = 0.010; *Bax*:*Dscaml1*, p < 0.001). Abbreviations: WT = wildtype, RBC = rod bipolar cell. Asterisks indicate significance. Arrowheads = dendritic tips. Sample sizes = WT: 8 RBC, *Bax*: 6 RBC, *Dscaml1*: 6 RBC. Error bars = standard error of the mean. Scale bar = 5 μm.

We next counted the number of dendritic tips of individual RBC dendritic arbors in wildtype, *Bax* null, and *Dscaml1* mutant retinas (**Fig 5A–5C**). We simultaneously counted the number of dendritic tips that terminated within the rod spherule. We observed several examples of a single rod being innervated by multiple RBC dendrite tips originating from the same cell in the *Dscaml1* mutant retina, something we did not observe in other genotypes (**Fig 5D**). We found a reduction in the number of total RBC dendritic tips in both the *Bax* null and *Dscaml1* mutant retina compared to wildtype, and no difference between the number of dendritic tips formed by *Dscaml1* mutant RBCs when compared to the number of *Bax* null RBC dendritic tips (WT:*Bax*, p = 0.019; WT:*Dscaml1*, p < 0.001; *Bax*:*Dscaml1*, p = 0.37) **(Fig 5E**). We divided the number of dendritic tips that invaginated into rod spherules by the total number of dendritic tips to compare the percentage of total dendritic tips that contacted rod spherules across the wildtype, *Bax* null, and *Dscaml1* mutant retina. We traced wildtype and *Bax* null RBC dendritic tips to the rod spherule 86.9% and 91.0% (respectfully) but were able to trace only 63.5% of *Dscaml1* mutant dendritic tips to the rod spherule (WT:*Bax*, p = 0.350; WT:*Dscaml1*, p = 0.010; *Bax*:*Dscaml1*, p < 0.001) (**Fig 5F**).

### Mitochondrial volume and complexity are reduced in *Dscaml1* mutant retina compared to controls

**Fig 6:**
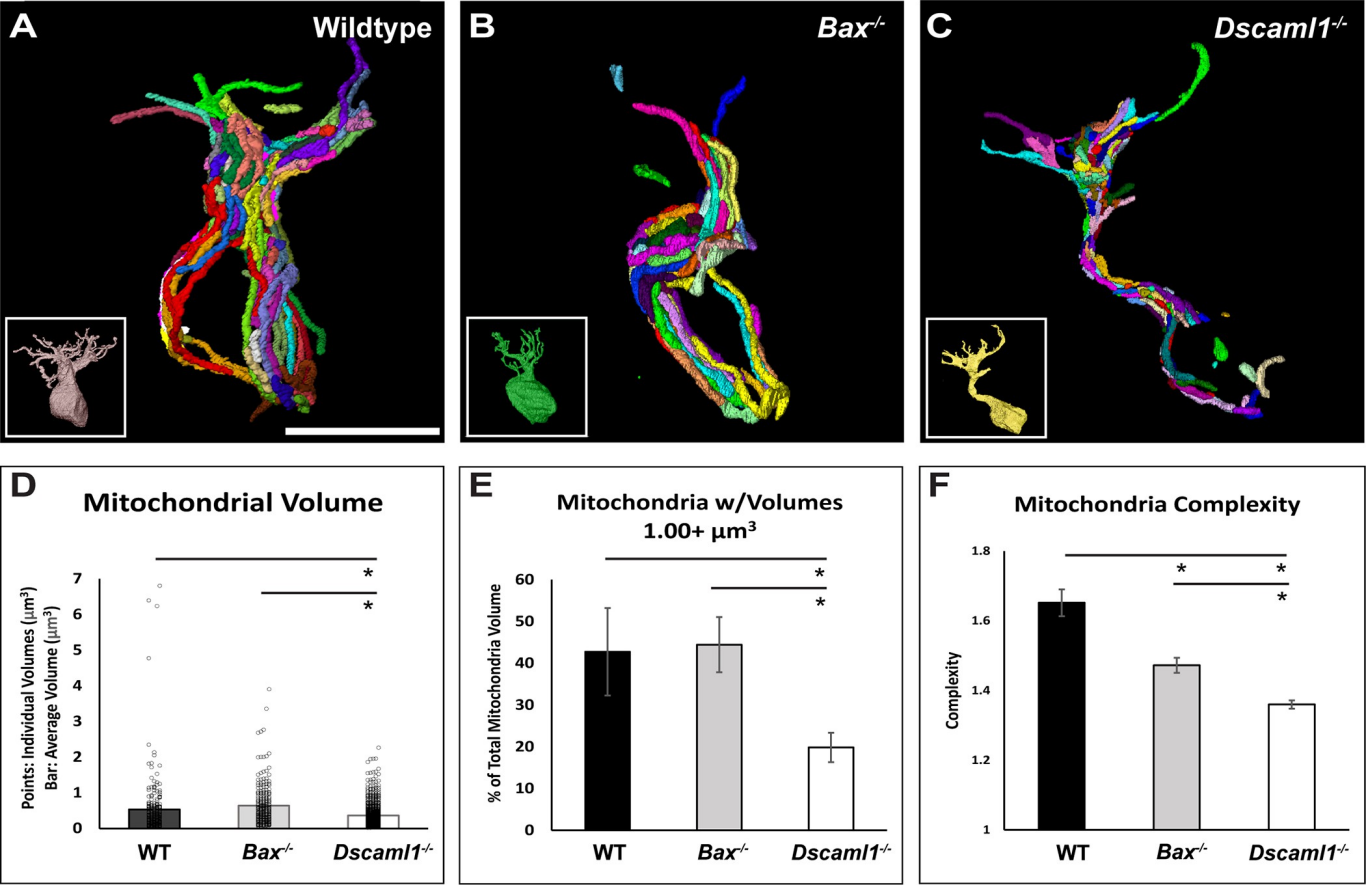
3D reduced mitochondria size and complexity in the *Dscaml1 mutant* retina. **A)** 3D reconstruction of mitochondria in RBC of the wildtype retina. **B)** 3D reconstruction of mitochondria in RBC of the *Bax* null retina. **C)** 3D reconstruction of mitochondria in RBC of the *Dscaml1* mutant retina. **D)** A decrease in the average mitochondrial volume was observed in the *Dscaml1* mutant RBC soma and dendrites when compared to both wildtype and *Bax* null retinas. (p = 0.002 and p < 0.001, respectfully). **E)** A decrease in the percent of total mitochondrial volume that mitochondria over 1.00 um^3^ make up in *Dscaml1* mutant retina was observed compared to wildtype and *Bax* null retinas (p = 0.043 and p = 0.031, respectfully). **F)** A decrease in mitochondrial complexity was observed in both the *Bax null* and *Dscaml1* mutant RBC when compared to wildtype retina (p = 0.006 and p < 0.001), with a larger decrease in complexity in *Dscaml1* mutant compared to *Bax* null retina. (p < 0.001). Each color represents individual mitochondria. Abbreviations: WT = wildtype. Asterisks indicate significance. Mitochondria sample sizes (for all data) = WT: 234, across 3 RBC; *Bax*: 235, across 3 RBC; *Dscaml1*: 684, across 6 RBC. Error bars = standard error of the mean. Scale bar = 5μm.

We measured the size, volume, and complexity of mitochondria in RBCs. Dscam genes regulate developmental cell death through Bax-dependent mitochondrial developmental cell death, and we were curious if there were ultrastructural differences at the mitochondria [13, 23, 55]. We compared individual volumes of mitochondria within RBCs of wildtype, *Bax* null, and *Dscaml1* mutant retinas (Fig 6A–6C). We observed a reduced average mitochondrion volume in *Dscaml1* mutant RBCs when compared to both wildtype and *Bax* null RBCs (p = 0.002 and p < 0.001, respectfully) **(**Fig 6D). We also noticed that the percentage of larger mitochondria was reduced in the *Dscaml1* mutant retina compared to controls (Fig 6E). Mitochondria with volumes greater than 1.00 μm^3^ contributed to 42.8% and 44.5% of the total mitochondrial volume within wildtype and *Bax* null RBCs (respectfully), compared to only 19.3% of total mitochondrial volume in *Dscaml1* mutant RBCs (WT:*Bax*, p = 0.910; WT:*Dscaml1*, p = 0.043; *Bax*:*Dscaml1*, p = 0.031) (Fig 6E). Increased mitochondrial function can be correlated with increased complexity and fusion of the mitochondria within a cell [56, 57]. We therefore also calculated the complexity of each mitochondrion using a surface area to volume ratio that allowed us to determine how far a mitochondrion strayed from a perfect sphere with a ratio of 1:1. We found that mitochondria in both *Bax* null and *Dscaml1* mutant retinas had reduced complexity when compared to wildtype mitochondria (p = 0.006 and p < 0.001) (respectfully), with *Dscaml1* mutant mitochondria showing even less complexity than *Bax* null RBC mitochondria (p < 0.001) (Fig 6F).

## Discussion

In this study, we measured anatomical formation and maintenance of the rod synapse in wildtype and *Dscaml1* mutant mice. A mutation in *Dscaml1* has been linked to middle age onset of congenital stationary night blindness. *Dscaml1* is expressed in cells of the rod circuit of the mouse retina, consistent with this finding. We used a loss of function mouse model to measure formation and anatomical connectivity of the synapse during the mouse life span to test if *Dscaml1* is required to maintain the rod synapse. We identified changes in connectivity, aging and organization of the synapse.

The rod circuit is susceptible to different degrees of age-related degeneration and changes in different species. Loss of rods, RBCs and sprouting of RBC dendrites associated with aging have been reported in humans, while other animals, such as marmosets do not appear to undergo age related changes to the rod circuit, or even zebrafish, which continuously produce rods during the lifespan [27–32, 58]. Mice represent an intermediate between these examples with evidence of rod denervation and RBC dendrite sprouting but not significant cell loss. We observed a similar gross organization pattern to the wildtype in the aging *Dscaml1* mutant mouse retina. RBC and horizontal cell neurite sprouting into the outer nuclear layer is associated with both aging in the mouse retina and synaptic dysfunction [15, 16, 31]. We did not observe excess sprouting and synapse formation during early developmental stages or advanced age (1 year and 18-months) but did observe an increase in sprouting at an intermediate time point in the *Dscaml1* mutant retina, suggesting that normal processes observed in the aging retina may be advanced in the absence of *Dscaml1*. An increase in RBC sprouting has been observed in mutant mouse models lacking ribbon and other synaptic proteins, and while *Dscaml1* is not required for the formation of functional synapses [13, 14, 16, 40–43], its absence did result in precocious sprouting in this study, suggesting a role at the rod synapse.

Next, we measured the ultrastructure of the rod synapse and bipolar cell dendritic fields using serial electron micrograph reconstructions. We found that the number of invaginations was increased in *Dscaml1* mutant retinas compared to controls (Fig 3). We observed overlap of RBC dendritic arbors in all genotypes, consistent with the known lack of RBC dendritic arbor tiling. We did not observe significant clumping or fasciculation of RBC dendrites with each other like has been observed for other cell types, but this observation may have been limited by the number of cells traced per field and the density of RBCs. Cone bipolar cells exhibit a form of plasticity after loss of *Dscam* that involves sprouting of dendrites [24]. We observed a reduced number of dendrite tips in the *Dscaml1* mutant retina and an increase in the number of tips that do not make anatomical connections with rod spherules, which would be consistent with dendritic sprouting observed in OFF bipolar cells in the *Dscam* mutant retina. In the wildtype retina, RBCs occasionally make synaptic connections at cone pedicles. Compared to other studies, we observed a higher percentage of wildtype RBC dendrite tips that did not terminate at the rod spherules and speculate that this may be due to the slice thickness of the serial electron micrograph stacks used in this study compared to other studies in which less tissue was removed between serial image acquisition. We also observed an increase in the number of invaginating neurites in rod spherules, despite a decrease in the number of dendritic tips per RBC, compared to wildtype and *Bax* null retinas. Contacts between cone bipolar cells and rod spherules have been observed in other mutant genetic backgrounds and may be occurring here also, but thorough tracing of stacks will be required to determine if additional invaginations observed at rod spherules, despite the decrease in RBC tips, can be explained by other cell types contacting RBCs in the *Dscaml1* mutant retina [59]. Different Dscam1 isoforms are known to interact weakly in *Drosophila* and related sidekick proteins also interact weakly with each other [25, 60, 61]. Similar weak interactions between *Dscam* and *Dscaml1* could contribute to rod-cone pathway specificity. Additional volume generation and tracing can be performed to test this. We also observed several instances in which RBC dendrite tips branched immediately before both tips invaginated into the same rod spherule in the *Dscaml1* mutant retina (Fig 5D) but not in other genotypes, suggesting an iso-neuronal role for *Dscaml1* in RBC, similar to iso-neuronal avoidance mediated by *Drosophila* [62, 63]. Other studies have reported a change to the rod ribbon ultrastructure in aging mice [64]. EM studies at more advanced ages could help to determine if similar changes occur in *Dscaml1* mutant mice.

We also measured mitochondria in wildtype, *Bax* null, and *Dscaml1* mutant mouse RBCs and observed a decrease in both the individual volume and complexity of the mitochondria in *Dscaml1* mutant RBCs compared to controls. Mitochondria in neurons are involved in many intracellular processes beyond ATP production and are essential to proper signal transduction through nuclear gene regulation, metabolism, synaptic vesicles, reactive oxygen species production, and ion regulation [65–69]. Mitochondria accomplish this through their ability to undergo morphological changes: fusion and fission [70]. Larger mitochondria increase ATP yields and calcium homeostasis, while smaller mitochondria are involved in intracellular transportation, observed at apoptosis initiation, and often are targeted to undergo mitophagy to improve cellular stability [56, 57, 71–75]. However, fragmentation and decline in mitochondrial quality has also been associated with mitochondrial dysfunction, contributing to the severity of neurological diseases, disorders, and aging [76–81]. These findings demonstrate that intracellular factors may contribute to individual RBC function that is not detectable by a measure of bulk output like electroretinogram, especially in cases where there is an increase in RBC number that can disguise ERG results. However, it is unclear if changes in the mitochondria are primary or secondary to changes in dendritic organization in RBCs. A further limitation is that while a large number of mitochondria were reconstructed, these were sampled from a small number of RBCs. Additional tracing will establish the degree of variability of mitochondria within RBCs in addition to across genotypes.

In conclusion, we surveyed the rod-to-RBC synapse in *Dscaml1* mutant mouse retina from postnatal development through aging and observed that synapses are formed and maintained like what is observed in wildtype mouse retina. We measured the number of neurite invaginations at the rod spherule and found an increase in the number of invaginations in *Dscaml1* mutant retina compared to controls. We quantified the number of dendritic tips in wildtype, *Bax* null, and *Dscaml1* mutant retina. Both the total dendritic tip number and percent of dendritic tips that contacted spherules was reduced in *Dscaml1* mutant retina compared to controls. Finally, we measured individual mitochondrial volume and complexity in RBCs of wildtype, *Bax* null, and *Dscaml1* mutant retina and report a decrease in both the mitochondrial volume and complexity when compared to controls. The rod circuit differs significantly in mouse and human. Rods represent the vast majority of photoreceptors in mice but have a sparse distribution in humans. It is possible that changes shown here are sufficient to further degrade the minimal human rod pathway. Mutations observed in humans are missense compared to the loss of function mutation used here and this is a known limitation of this study. However, these results contribute knowledge of *Dscaml1’s* role in rod circuit development and maintenance and give insight into possible genetic therapy targets for blinding diseases and disorders like CSNB.

## Supporting information

S1 Dataset (XLSX)
